# Imposition of encapsulated non-indigenous probiotics into intestine may disturb human core microbiome

**DOI:** 10.3389/fmicb.2014.00393

**Published:** 2014-07-31

**Authors:** Abolfazl Barzegari, Solat Eslami, Elham Ghabeli, Yadollah Omidi

**Affiliations:** ^1^Student Research Committee, Research Center for Pharmaceutical Nanotechnology, School of Advanced Biomedical Sciences, Tabriz University of Medical SciencesTabriz, Iran; ^2^Student Research Committee, School of Advanced Technologies in Medicine, Tehran University of Medical SciencesTehran, Iran; ^3^Student Research Committee, Faculty of Medicine, Urmia University of Medical SciencesUrmia, Iran; ^4^Research Center for Pharmaceutical Nanotechnology, School of Advanced Biomedical Sciences, Tabriz University of Medical SciencesTabriz, Iran

**Keywords:** metagenome, microbiome, encapsulation, probiotic

## The cross-talk between probiotic bacteria and human host

The human health is deemed to be maintained by cross-talk among the body, probiotic and even pathogenic bacteria within the intestine, even though the precise molecular mechanisms governing such intercommunication remain to be fully understood (Corthésy et al., 2007). The human intestinal tract encompasses one of the most diverse microbial settings ever discovered. In the human intestinal lumen, the number of bacteria is approximately 10 times higher more than the number of intestinal cells. These microorganisms adapted to their human host are the natural beneficial symbionts of human host and hence are regarded as the second human genome (Saei and Barzegari, 2012).

It should be highlighted that the human genome is highly stable during lifetime. However, dependent on the life style and/or types of nutrition, human metagenome could be subjected to some minor and major alterations with possible implications in modern diseases such as asthma, allergies, cancers, and obesity (Eslami et al., 2012). Thus, any disruption in the inherent nature of the GIT milieu can inadvertently have unfavorable and irreparable even tragic consequences (Saei and Barzegari, 2012).

Today, bacteria known as probiotics are used to maintain the microbial balance of the GIT ecosystem—a notion which was first suggested by Nobel laureate Metchnikoff. Though *in vitro* tests have widely been used for studying the probiotics, correlation of *in vitro* and *in vivo* findings appears to be somewhat problematic. Although these studies provide valuable information on the human microbiome, the adaptive co-evolution of natural microbiome and its functionalities in human health and diseases are yet to be fully studied. It seems that the “Human Microbiome Project” will help scientists answer these important questions (Turnbaugh et al., 2007), nevertheless the functional emergence of microbiome appears as complex systems with co-op networks in co-adaptation with bio-components of GIT milieu. Despite all these issues, some health benefits have been observed for several probiotics, whose deciphering mechanism(s) of action can be of utmost significance. Perhaps, one of the major modes of action of the probiotics is through modulation of the host immune system, as they are hypothesized to invigorate the intestinal immune barriers (Forsythe and Bienenstock, 2010). The immune-enhancing effects obtained from probiotics may be attributed to (a) enhancement of secretion of immunoglobulin A (Parvez et al., 2006), (b) stimulation of pro-inflammatory pathway, (c) regulation of interleukins (ILs), and (d) activation of cytokine pathways (Parvez et al., 2006).

The human intestine carries trillions of bacteria including 400 different bacterial species. Given that the microbial balance is one of the key factors in human health, maintaining the delicate balance of the intestinal microflora is vitally important. Correspondingly, any imbalance among intestinal natural microflora and virulent bacteria may inevitably result in emergence of illness(s) (Barzegari et al., 2014).

## Probiotic therapy and controversial results

People have used probiotics for many years in the form of fermented dairy products such as yogurt, kefir and milk. Accordingly, after the first introduction of health benefits of probiotics in the early 12th century by Nobel Laureate Metchnikoff, many bacterial strains have clinically been tested as potential probiotics. Until now, reasonable evidences of human studies and meta-analyses have indicated the benefits of probiotics in a variety of conditions, including shortening the rotavirus diarrhea (Marteau et al., 2001), therapy of irritable bowel syndrome (Lee and Bak, 2011), reducing the recurrence of bladder cancer (Feyisetan et al., 2012), reducing the infection of *Clostridium difficile* (Hell et al., 2013), lowering the plasma cholesterol (Hell et al., 2013), preventing the necrotizing enterocolitis in preterm neonates (Deshpande et al., 2010), and preventing and treating the pouchitis and atopic dermatitis in children (Rautava et al., 2012). However, most of these effects appear to be strain- and/or population-specific (Barzegari and Saei, 2012). Why do not all studies confirm the benefits of probiotic therapy in particular conditions? This is an intrigue question that needs to be addressed. It seems the controversial results might be related to the dosages of probiotics and concentration of bacteria used in the probiotic supplements and diets (e.g., industrial fermented milks with narrow biodiversity of microbe starter culture) and encapsulated forms of human non-coevolved and/or non-native probiotics (Barzegari and Saei, 2012).

## Encapsulated probiotics disturb human core microbiome

The human intestine is an anaerobic bioreactor which contains a large bacterial setting composed of hundreds of species and thousands of subspecies (Xu et al., 2007). The number of bacterial cells in human body is profoundly greater than the number of human cells. Where have these bacteria come from? Upon delivery, when the immune system and gastrointestinal system have not evolved yet, the newborn baby exposes to a wide volume of environmental and maternal microbes (Dominguez-Bello et al., 2010). These microbes are also claimed for evolution of immune as well as gastrointestinal systems (Arboleya et al., 2012), but which microbes (indigenous symbiotic bacteria or non-indigenous ones) are fortunate enough to colonize the intestine? Since the harsh conditions of gastrointestinal system is not developed in infants and the immune system is not evolved yet, all types of bacteria have the same chance to enter the infants' gastrointestinal system. Upon delivery especially vaginal delivery, infants are exposed to vaginal microbes and gradually they will expose to further number of microbes through breast-feeding and the selection of symbiotic bacteria will be made as well (Harmsen et al., 2000). It has been demonstrated that infants born by Cesarean section (CS) represent differences in their microbiota in comparison with vaginally born infants (needs reference) and correspondingly CS delivery infants expose different diseases particularly allergies (Dominguez-Bello et al., 2010). With the evolution of immune system and its interaction with colonized florae and the harsh condition present in gastrointestinal system, only a particular number of bacteria will get the certificate to colonize the intestine. Indeed the immune system and gastrointestinal system are coevolved with normal microflora (Arboleya et al., 2012) and the interaction among those guarantees the health of human (Barzegari et al., 2014). Any interference in this homeostasis through the encapsulation of non-indigenous bacteria and their exposure disturbs the dominant balance and exposes the person to diseases.

These bacteria are now considered as the human second genome, and have been shown to contribute to the human health by (a) the modulation of immune system, (b) the exclusion of the pathogens, and (c) the association in the digestion process. It should be highlighted that real probiotic bacteria are those that can well tolerate and survive the GIT transit, colonize, and eventually become metabolically active members of the human intestine. To the best of our knowledge, ignoring the high colonization potential of the bacterial probiotics and administering them in high concentration, despite possessing some favorable probiotic characteristics but lacking the survival traits and persistence capabilities, may disturb the evolutionarily-developed intestinal balance and in time give rise to some serious inadvertent imperceptible side effects. Intestinal microflora should be considered as complex systems. Hence, even trivial alterations in microflora of intestine accumulated in a long-term process may elicit drastic changes within the composition and setting of intestine, which can in return impact the intestinal microbiome biofunctions and metabolomes as well as human normal physiology in a holistic manner.

**Figure 1:**
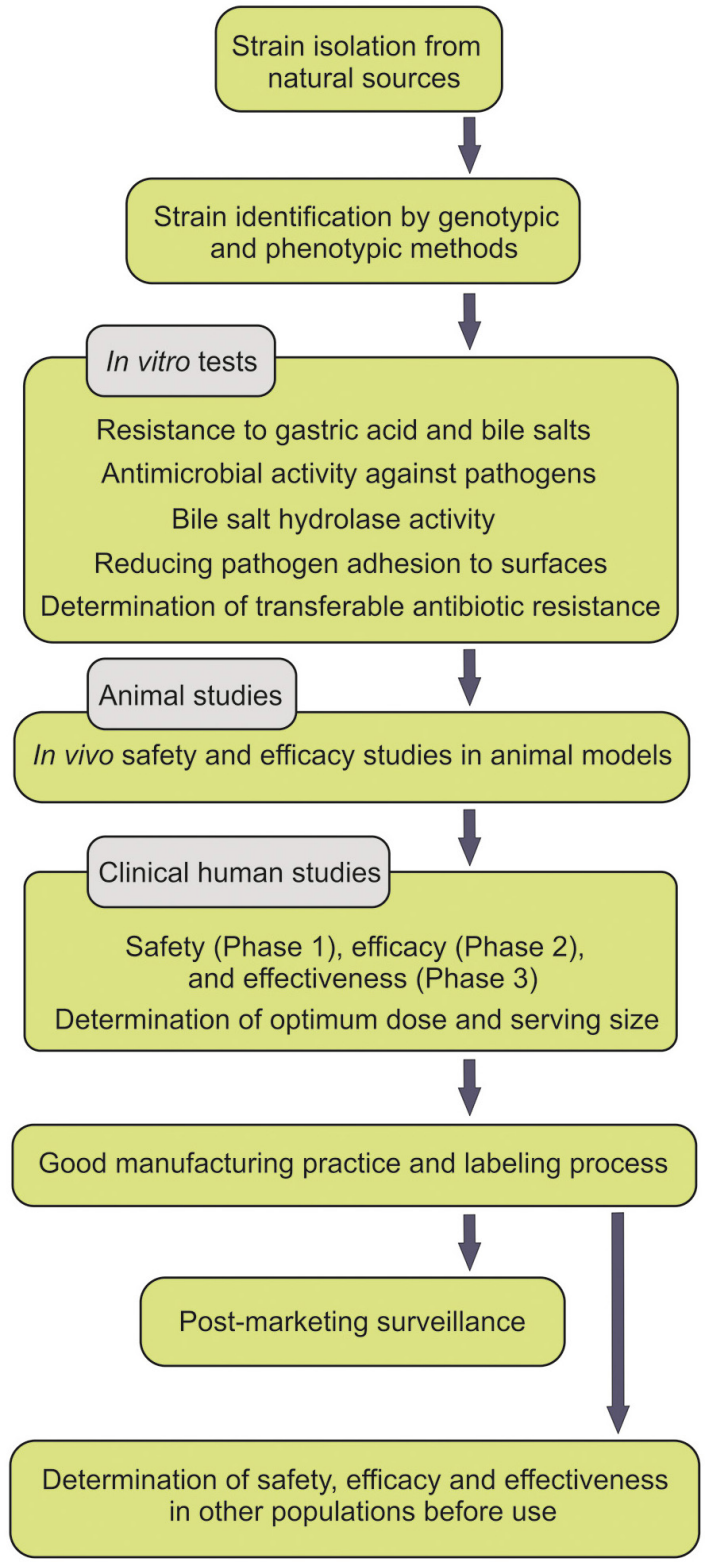
**WHO Guideline for isolation and application of probiotics**.

People pay a lot for probiotic supplements, but benefit little. Moreover, the safety of probiotics has sometimes been argued to be in part dependent upon the intestinal microbiota and the status of the host immune system (Gronbach et al., 2010). Some probiotics have been shown to transit the GIT intact with no persistence. Further, the spurious probiotics appear to be undetectable after the termination of the consumption course of the probiotics. Some studies convincingly demonstrated that most ingested strains fail to become persistent members of the intestinal microbiota, while solely some stains show short-term persistent viability after the course of the administration (Klingberg and Budde, 2006). For instance, *L. reuteri* was reported to be detectable solely in four and two (out of nine volunteers examined) respectively 1 and 2 weeks after the termination of the probiotic administration (Smith et al., 2011). All experts in the field agree that persistent viability of the administered probiotics within the GIT is a determining indicator for the efficacy of probiotics, however the health promoting outcomes may be intriguingly challenging. All these happen because the isolated strains, used as probiotics, are not either from the human origin, indigenous or even not carefully selected according to the criteria set forth by FAO/WHO guidelines (Figure 1).

As stated before, indigenous probiotic bacteria can tolerate the harsh conditions of the GIT (Iyer et al., 2005), while non-indigenous bacteria are relatively intolerant to such harsh conditions. Even if the encapsulation has shown to enhance the survival rate of probiotics within the GIT milieu, prior to their administration, some important unclear issues need to be fully addressed. Are not supposed the probiotic bacteria to be resistant to the GIT conditions as disclosed in FAO/WHO guidelines and analyzed by *in vitro* acid and bile tests? If so, why should one attempt to encapsulate the probiotics in order to enhance their viable delivery into the intestine?

To protect the viability of the probiotic bacteria during transition into the intestine, several methods of encapsulation (e.g., spray drying, extrusion, emulsion) have been developed using different types of biopolymers such as alginate, gelatin, chitosan, and cellulose derivatives (Huq et al., 2013; Shi et al., 2013; Tee et al., 2013). Probiotic encapsulation is not always under question because it can enhance the survival rate of probiotics, increase their tolerance during food production processes and lyophilization, and preserve their viability in long-term storage (Huq et al., 2013).

However, the encapsulation process may elicit undesired impacts by introducing a high number of inappropriately-selected bacteria into the intestine and hence inducing inevitable interference with the natural intestinal ecosystem. Furthermore, the concept of viable probiotics can sometimes be questionable, since dead probiotic cells have also been shown to induce some biological responses mainly by the immune system of the host (Adams, 2010). The symbiosis of intestinal microbiota with the human intestine has occurred over many millennia of adaptive coevolution, leading to a complex biological and reciprocal relationship between bacterial and *Homo sapiens* cells (Ley et al., 2008). Thus we cannot expect an outlander, a bacterium which does not belong to this setting, or one that has not been able to live within such symbiosis with human intestine through the many years of coevolution, to serve as a probiotic. Therefore, unknown interactions of these pseudo-probiotics can lead to possible alterations in human metagenome. If these bacteria were favorable to their human host, they would not be filtered by the defensive mechanisms of GIT. Hence, encapsulation of probiotics for enhancing their survival rate during GIT transition looms to be a matter of rigorous scientific debate. In fact, “to think that we can intervene effectively in human–microbe relationships without considering microbial ecology and evolution” is not a scientifically sound approach (Dethlefsen et al., 2007).

Since the probiotic-intestinal machineries are very sensitive at molecular levels and also the immune system can be modulated by even small amounts of an antigen, the nature and the number of bacteria in probiotic supplements should necessarily be revisited based upon the natural setting of the intestine. To benefit the consumer, the minimum recommended number of viable bacteria in food products has been suggested to be 10^7^ CFU per mL at the time of consumption, while lower or higher number of bacteria may be demanded for a specific biological function. This issue again can be dependent on (a) the strain used, (b) the intestinal microbial balance, and (c) the pathophysiologic condition that is going to be treated. The large intestinal microbiome species (Xu et al., 2007) is indeed an interwoven complex bionetwork composed of trillions of interactions. Then, imposing high numbers of only one specific probiotic may upset the equilibrium of intestinal microbiome or change it in favor of certain strain(s). It seems that the recommended number of probiotic bacteria (10^7^ CFU per mL), which should exist in probiotic preparations, comes without a scientifically sound basis and seems to be somewhat over-generalized approach that may not visit all facts required for all probiotic strains and for all the purposes of the administration.

## Discussion

The harsh conditions of GIT (i.e., the presence of acid, pepsin, bile salts and pancreatic secretions) hinder majority of the ingested microorganisms to enter the intestine and also protect human body against the undesired interfering bacteria (Schwabe and Jobin, 2013). Only a small portion of these microorganisms co-evolve with human intestine in a way that they can tolerate such hostile environment and build the naturally adoptive intestinal microbiome. Thus, the balance of the intestinal ecosystem is determined/maintained by a robust appointive/selective process imposed by the GIT environment, human genetics and lifestyle (Schwabe and Jobin, 2013). The importance of such selective microbiome is extremely complex and immense, which is regarded as human second genome/metagenome. Having considered these facts, any irrational manipulation of the intestinal microbiome can have undesired and sometimes irreversible harsh consequences. In addition, the intestinal dysbioses is associated with a variety of modern diseases such as malignancies (in particular colon cancer), diabetes, ulcerative colitis, inflammatory bowel disease, and multisystem organ failure (Cho and Blaser, 2012; Schwabe and Jobin, 2013). Further, introduction of non-indigenous probiotics into the human intestine can lead to some alterations in the microbiome and metagenome, resulting in emergence of metabolic syndrome, autoimmune diseases, and probably epigenetic disorders in a short- or long-term period. Moreover, even accumulation of large numbers of indigenous probiotics can disrupt the complex and dynamic ecosystem of the intestine. In fact, little is known about the intestinal microbiome and its cross-talk with the human intestine, hence great care should be taken into account when selecting probiotic bacteria for an individual with certain health condition—perhaps we should consider probiotic supplements as personalized medicines.

To conclude, we envision that multi-probiotic therapy and holistic personalized microbiome supplements may be scientifically justified and valued in individualized probiotic designs and even targeted therapy of life-threatening diseases such as cancers in the future. Subsequently, consumer health settings and advancements in science, all should specially be attended in the probiotic design modalities and strategies, rather as personalized medicaments. Although the guidelines imposes extra costs and labor, and time to characterize good probiotics, the isolation of real indigenous, beneficial bacteria may also provide some advantages. Respecting the scientific disciplines has the potential to increase the public awareness about the health benefits of real probiotics. How nice it would be if we could imagine a future in which probiotics are incorporated in our drug pharmacopeias with their associated health benefits! In such a future, people could truly believe in probiotics, and probiotics could even be considered and consumed as personalized medication.

### Conflict of interest statement

The authors declare that the research was conducted in the absence of any commercial or financial relationships that could be construed as a potential conflict of interest.
